# HIV-associated sensory neuropathy in HIV-1 infected patients at the Douala General Hospital in Cameroon: a cross-sectional study

**DOI:** 10.1186/1742-6405-9-35

**Published:** 2012-11-26

**Authors:** Henry Namme Luma, Benjamin Clet Nguenkam Tchaleu, Marie Solange Doualla, Elvis Temfack, Victor Nicolas King Sopouassi, Yacouba Njankouo Mapoure, Vincent-de-Paul Djientcheu

**Affiliations:** 1 Department of internal Medicine, Douala General Hospital, Douala, Cameroon; 2 Department of Neurosurgery, Yaoundé Central Hospital, Yaoundé, Cameroon; 3 Faculty of Medicine and Biomedical Sciences, University of Yaoundé 1, Yaoundé, Cameroon; 4 Université des Montagnes, Bagangte, Cameroon

**Keywords:** HIV-associated sensory neuropathy, HIV, CD4 count, Peripheral neuropathy

## Abstract

**Background:**

Peripheral neuropathy (PN) which is the most common neurological complication of HIV infection is under recognised and undertreated especially in resource limited settings. This ailment which has a negative impact on the quality of life of HIV/AIDS patients exists in different clinical patterns of which HIV-associated Sensory neuropathy (HIV-SN) is the most common affecting up to two thirds of patients with advanced disease in some settings. In Cameroon where HIV is a major public health problem, the burden of HIV-SN has not yet been well defined.

**Methods:**

Using the Brief Peripheral Neuropathy Screening (BPNS) tool validated by the AIDS Clinical Trial Group (ACTG) we carried out a cross sectional study to determine the prevalence of HIV-SN and its associated factors among HIV-1 patients at the Douala General Hospital between 1^st^ July and 31^st^ October 2011. HIV-SN was defined as the presence of neuropathic symptoms and at least an abnormal perception of vibrations of a 128Hz tuning fork on the great toe or abnormal ankle reflexes or both and expressed as a percentage of the study population.

**Results:**

Out of 295 patients studied, 21% had HIV-SN. In HIV-SN patients the median duration of HIV infection was 79.8 months (IQR 46 – 107.5) and their median CD4 count 153cells/μL (IQR 80 – 280). Patient recall and clinical chart review showed that, 83.9% had neuropathic symptoms prior to HAART initiation and 16.1% after HAART initiation. Low CD4 count, history of alcohol intake and history of anti-tuberculosis treatment were strongly associated with HIV-SN (AOR 2.5, 2.8 and 2.9 respectively).

**Conclusions:**

HIV-SN is common among patients with advanced HIV infection in Cameroon. This simple diagnostic tool (BPNS) should therefore be routinely used to detect those with HIV-SN or at risk so as to minimise the negative impact it has on their quality of life.

## Background

Peripheral neuropathy (PN) which is the most common neurological complication in HIV infection [1] is widely under-recognised and under treated in resource constrained settings [2]. Clinically, there are at least six patterns of HIV associated peripheral neuropathy; distal sensory polyneuropathy (DSP), inflammatory demyelinating polyneuropathy (IDP), progressive polyradiculopathy (PP), rapidly progressive flaccid paraparesis, mononeuritis multiplex (MM), and autonomic neuropathy [3]. Distal sensory peripheral neuropathy which is the most common of all HIV-associated sensory neuropathy (HIV-SN) [4] exists as two major types: primary HIV-associated distal sensory polyneuropathy (HIV-DSP), and antiretroviral toxic neuropathy (ATN), ATN being the most frequent antiretroviral therapy related toxicity in sub-Saharan Africa [5-8]. HIV-DSP and ATN together involve approximately 30-67% of patients with advanced HIV disease [1]. Despite significant improvement in the overall health of HIV-infected patients in the highly active antiretroviral therapy (HAART) era, HIV-SN still remains an important cause of morbidity among these patients because it considerably affects their quality of life [9,10]. The presence of HIV-SN-related neuropathic pain is a factor associated with greater unemployment, higher rates of depression and greater dependency in daily life activities albeit successful HAART [9]. More so, symptomatic relief therapy is usually not satisfactory [11]. In sub-Saharan Africa, many studies have not used a validated diagnostic tool to diagnose HIV-SN. In Cameroon, where the prevalence of HIV among adults has been estimated to be 5.3% [12] the burden of HIV-SN has not yet been estimated. Furthermore, HAART is still composed of first line drugs some of which have been demonstrated in other studies to increase the risk of PN [13,14]. We therefore, decided to determine the prevalence of HIV-SN and its associated factors in HIV infected patients at the Douala General Hospital using an easy but validated screening tool, the Brief Peripheral Neuropathy Screening (BPNS) tool [15].

## Results

### Patient characteristics

A total of 295 adult HIV-1 infected patients were included in the study, 69.8% (206/295) of who were females (Table 1). The mean age, weight and height were 42.3 ± 10.4 years, 70.6 ± 12.8kg, and 1.6 ± 0.1m respectively. The median duration of HIV infection by 31^st^ October 2011 was 71 months (IQR 34.4 – 101.5). The median CD4 cell count was 200cells/μL (IQR 102 – 300). Of the 295 patients, 96.9% (286) were on HAART, 45.4% (134/295) of who were on a regimen containing AZT-3TC-NVP (Table 1).

**Table 1:** **General characteristic of 295 patients with HIV 1 infection screened for HIV**-**SN**

**Characteristic**	**n** (%)
Age group	
<30	34 (11.5)
30 – 39	96 (32.5)
40 – 49	94 (31.9)
50 – 59	59 (20.0)
>60	12 (4.1)
Sex	
Male	89 (30.2)
Female	206 (69.8)
CD4 count	
<200	143 (48.5)
>200	152 (51.5)
CDC staging categories	
A1	8 (2.7)
A2	36 (12.2)
A3	31 (10.5)
B1	4 (1.2)
B2	73 (24.8)
B3	67 (22.7)
C1	6 (2.0)
C2	27 (9.2)
C3	43 (14.6)
HAART regimen	
AZT-3TC-EFV	46 (15.6)
AZT-3TC-NVP	134 (45.4)
TDF-3TC-EFV	22 (7.5)
TDF-FTC-EFV	4 (1.4)
D4T-3TC-NVP	46 (15.6)
Others	34 (11.5)
Not treated	9 (3.1)
History of anti-tuberculosis treatment	
Yes	31 (10.5)
No	264 (89.5)
History of alcohol intake	
Yes	33 (11.2)
No	262 (88.8)

AZT: Zidovudine, 3TC: Lamivudine, EFV: Effavirenz, NVP: Nevirapine, D4T: Stavudine, TDF: Tenofovir.

### HIV-associated sensory neuropathy (HIV-SN)

Using the BPNS tool, the prevalence of neuropathic symptoms was 28.5% (84/295) the most common of which was sensation of pins and needles on legs/feet (Table 2). The median grade of symptoms on a scale of 0 to 10 was 4 (IQR 3 – 6). Among patients with symptoms, 36.9% (31/84) had both abnormal reflexes and abnormal perceptions of tuning fork vibration, 19% (16/84) had only abnormal reflexes, 17.9% (15/84) had only abnormal perception of vibrations and 26.2% (22/84) had neither abnormal perception of vibration nor abnormal reflexes. Among those without symptoms, 27.5% (58/211) had at least an abnormal perception of vibrations or abnormal reflexes.

**Table 2:** **Prevalence of signs and symptoms consistent with HIV**-**SN**

**Symptoms** (**at the legs or feet**)	**n** (%)
Pain	21 (7.1)
Pins and needles	39 (13.2)
Numbness	24 (8.1)
Asymptomatic	233 (78.9)
**Ankle jerk reflexes compared to knee jerk**	
Grade 0 : Absence of reflex	41 (13.9)
Grade 1: Hypoactive	48 (16.3)
Grade 2: Normal	203 (68.8)
Grade 3: Hyperactive	2 (0.7)
Grade 4: Clonus	1 (0.3)
**Perception of tuning fork vibration on great toes**	
Grade 0: maximum perception for >10s	213 (72.2)
Grade 1: perception for 6 – 10 seconds	42 (14.2)
Grade 2: perception for <5 seconds	25 (8.5)
Grade 3: No perception	15 (5.1)

The prevalence of symptomatic HIV-SN was 21% (62/295) of the study population. Among patients with symptomatic HIV-SN, the median duration of HIV infection was 79.8 months (IQR 46 – 107.5) with a median CD4 count of 153cells/μL (IQR 80 – 280). Patient recall and clinical chart reviews, showed that 83.9% (52/62) had symptoms prior to HAART initiation with a median duration of symptoms of 24.3 months (IQR 10.8 – 44.7), the remaining 16.1% (10/62) developed symptoms after HAART initiation with a median onset of symptoms of 17.7 months (IQR 2 – 41).

### Association between HIV-SN and patient characteristics

HIV-SN was strongly associated with advanced age, low CD4 count, history of alcohol consumption, history of anti-tuberculosis treatment but was modestly associated with sex and height (Table 3). There was no association between HIV-SN and a history of D4T containing HAART regimens (Table 3).

**Table 3:** **Univariate analysis of possible associated factors for HIV**-**SN**

**Variable**	**HIV**-**SN**	**No HIV**-**SN**	**OR** (**95**% **CI**)	**P value**
Age				
<40	16 (25.8)	114 (48.9)	2.8	0.001
>40	46 (74.2)	119 (51.1)	(1.5 – 5.2)	
Sex				
Male	25 (40.3)	64 (27.5)	1.8	0.05
Female	37 (59.7)	169 (72.5)	(1 – 3.2)	
CD4 cell count				
<200	40 (64.5)	103 (44.2)	2.3	0.004
>200	22 (35.5)	130 (55.8)	(1.3 – 4.1)	
Height				
<1.7m	38 (61.3)	171 (73.4)	1.7	0.06
>1.7m	24 (38.7)	62 (26.6)	(1 – 3.1)	
History of alcohol intake				
Yes	13 (21)	20 (8.6)	2.8	0.006
No	49 (79)	213 (91.4)	(1.3 – 6.1)	
History of anti TB treatment				
Yes	11 (17.7)	20 (8.6)	2.3	0.04
No	51 (82.3)	213 (91.4)	(1.0 – 5.1)	
AZT in HAART regimen				
Yes	26 (41.9)	89 (38.2)	0.9	0.6
No	36 (58.1)	144 (61.8)	(0.5 – 1.5)	
D4T in HAART regimen				
Yes	12 (19.4)	34 (14.6)	1.4	0.4
No	50 (80.7)	199 (85.4)	(0.7 – 2.9)	
**Total**	**62** (**21**)	**233** (**79**)	/	/

After adjusting for age, sex and height, low CD4 count, history of anti-tuberculosis treatment and alcohol intake remained significantly associated with HIV-SN (Table 4).

**Table 4:** **Multivariate analysis of associated factors for HIV**-**SN adjusted for age**, **sex and height**

	**Adjusted Odd ratio** (**AOR**)	**95**% **CI**	**P value**
Low CD4 (<200/mm^3^)	2.5	1.3 – 4.6	0.003
History of alcohol intake	2.8	1.2 – 6.6	0.01
History of anti TB treatment	2.9	1.3 – 6.8	0.01

## Discussion

The definition of HIV-SN as per the BPNS tool assesses both subjective and objective findings consistent with PN. However, the requirements that a patient must have both neuropathic symptoms and clinical signs to be diagnosed as HIV-SN may exclude some patients with mild disease [16] indicating a substantial underestimation of PN [17]. This tool which is easy to use, practical and adds less than five minutes to the clinical examination of HIV patients during follow up in busy outpatient clinics [16] has been used in its standardised form in few studies. Using this tool, the prevalence of HIV-SN in our study population was 21% though, the prevalence of HIV-SN varies greatly among studies between 19 and 42% [16,18-22]. According to Skopelitis and collaborators who used a model which involved both clinical criteria (symptoms and signs of neuropathy) and electrophysiology studies, two thirds of HIV-SN are subclinical [23]. This therefore means that some patients though asymptomatic might have abnormal signs relevant to HIV-SN but which do not meet up with HIV-SN diagnostic criteria as per the BPNS tool. This was the case in 19.7% (58/295) of our study population. Some authors [19,20] considered this group as asymptomatic PN who perhaps represent those with early HIV-SN who are more likely to become symptomatic when challenged with HAART or other risks for PN [20]. Considering together those with HIV-SN (according to BPNS criteria) as symptomatic PN patients and those with clinical abnormalities relevant with PN but no symptoms as asymptomatic PN patients, increases the prevalence of PN in our study to 40.7%. In a resource limited setting like ours, this approach increases the likelihood of detection of patients with or at risk of PN. Consequently, caregiver choice of drugs of the HAART regimen would take into consideration each patient’s risk of PN and patient education on the avoidable risk factors would be accentuated. Either way HIV-SN is common among patients with HIV in Cameroon.

We found that HIV-SN is more common among older patients, similar with findings in other studies [4,16,19-21,24]. This is because peripheral nerves by their length and size are known to have increased vulnerability with aging due to continual metabolic stress and exposure to toxic substances of which alcohol is among the most common as could be seen in our study where after adjusting for age, sex and height alcohol intake still remained strongly associated with HIV-SN consistent with findings in similar settings like ours [22]. Morgello and collaborators in America also found that HIV-SN correlated with alcohol intake. Furthermore, increased life expectancy in the HAART era predisposes to longer exposure to the virus and/or to dideoxynucleoside analogues [23]. Even though, there is considerable evidence on the risk of PN in the HAART era with use of neurotoxic agents [4,17,25], our study failed to show any such association especially with D4T. Another study [19] found that pre-existing PN before starting HAART reduced the risk that a patient would be placed on a D4T based regimen [19] thereby iterating the importance of pre-HAART screening for PN. However, the absence of association between D4T use and PN in our study could have been because only 15.6% of our patients were on a D4T containing regimen compared to as much as 98% in other studies in similar limited resource settings [19,21].

A history of anti-tuberculosis treatment was found to be strongly associated to HIV-SN in our study. Isoniazid which is a major component of antiTB treatment during the whole treatment period has been shown to be a risk factor of PN [1].

Finally, our finding of low CD4 count, a reflection of advanced HIV disease, to be strongly associated with HIV-SN, was in line with findings in many other studies [4,16,17,19,21,24]. Moreover, given that 83.9% of our symptomatic patients had symptoms before HAART initiation could be evidence that HIV-SN primarily might have been be due to the virus (HIV-DSP) [20].

Our study had some limitations. Firstly being a hospital based study in a tertiary institution where care is expensive; there is possibility of selection bias not permitting us to capture the real picture of HIV-SN among HIV patients in Cameroon. Secondly, a longitudinal design, unlike our cross-sectional design would enable us determine the incidence of ATN most especially as HAART coverage is being scaled up.

## Conclusions

HIV-SN is common among HIV 1-infected patients at the Douala General Hospital. Simple neurological evaluation tools to detect early HIV-SN or those at risk among all HIV patients should be routinely used to diagnose this ailment which negatively impacts their quality of life. HIV-SN increases in frequency with severity of immune depression thereby necessitating early diagnosis and treatment of HIV to avoid severe immune depression. Finally, clinicians’ choice of drugs of the HAART regimen and other drugs for HIV comorbidities should consider the role these drugs play as a factor associated with HIV-SN most especially as we found that a proportion of our patients developed symptoms relevant with HIV-SN only after HAART initiation and use of anti-tuberculosis drugs.

## Methods

### Study setting and patients

After prior local institutional ethical clearance, we carried out a cross sectional study at the adult HIV outpatient clinic of the Douala General Hospital, a tertiary hospital with a capacity of 320beds, situated in Douala, the economic capital of Cameroon between 1^st^ July and 31^st^ October 2011. Our study population comprised of consenting adults (>18 years) diagnosed with HIV and followed up in this hospital. HIV diagnosis in this institution is made according to Cameroon National AIDS Control Programme guidelines [26] by antibody detection on two successive samples using a third generation ELISA test BIOREX® (Biorex Diagnostics Limited, Antrim, United Kingdom). If both are positive, a third sample is collected and tested using Genie® III HIV-1/HIV-2 Assay (Bio-Rad Diagnostics, Marnes la Coquette, France) to specify either HIV 1 or HIV 2. A patient is declared positive for HIV if these three tests are positive. In case of any discordance, testing is done with Western blot (New LAV blot, Diagnostics, Pasteur, Marnes la Coquette, France).

### Study procedure

Consecutively, each consenting patient underwent a brief peripheral neuropathy screening (BPNS) using the BPNS tool validated by the AIDS Clinical Trial Group (ACTG) [15]. This consists of brief questions regarding symptoms of HIV-SN: pain, aching or burning sensation in the feet/legs, pins and needles sensation in the feet/legs and numbness in the feet/legs. Screening was performed by a final year medical student trained for the purpose. Each symptom when present was subjectively graded bilaterally from 0 (absent) to 10 (severe). Lower extremity examination was then done to evaluate patient perception of vibrations for over 10seconds using a 128Hz tuning fork on the big toe. Ankle reflexes were also tested using a reflex hammer. Both findings were graded as follows: vibrations (grade 0: maximum perception for >10s, grade 1: 6 -10s, grade 2: <5s, grade 3: no perception) and ankle reflexes (grade 0: absent, grade 1: hypoactive, grade 2: normal, grade 3: hyperactive, grade 4: clonus). To ensure proper examination, random patients were chosen for examination by the neurologist whose findings were compared to those of the student for concordance and all were concordant. Socio-demographic and anthropometric information relevant to the study were also obtained from each patient and completed by clinical case files examination.

### Statistical analysis

Data entry was in Epi Data 3.1 and analysis in STATA 11.2 (Stata Corporation, College Station, Texas). HIV-SN was defined by the presence of symptoms and at least an abnormal perception of vibrations or abnormal ankle reflexes or both and expressed as a percentage of the study population. It was then categorised as either present or absent and compared to other variables using chi square test and Fisher’s exact test where necessary. Mantel Haenszel method was used to measure strength of association between HIV-SN and covariates and a final model was made using logistic regression. Crude and adjusted Odd Ratios (OR) together with its 95% Confidence Intervals (95%CI) was reported. Evidence of association was considered for a two-sided p value of less than 0.05.

## Competing interests

All authors declare no conflict of interest.

## Authors’ contributions

HNL, BCNT, MSD, ET and VPD designed the study. VNKS, BCNT, and YNM collected and entered data. HNL, BCNT and ET did data analysis and manuscript write-up. VPD and MSD did manuscript proofreading and editing. All authors agreed with the final manuscript to be submitted. All authors read and approved the final manuscript.
